# Translating Population-Based Epidemiology into Clinical Recognition of Coronary Artery Spasm: Prevalence, Incidence, and Geographic-Seasonal Variation in Taiwan

**DOI:** 10.3390/medsci14050582

**Published:** 2026-09-18

**Authors:** Alexander T. H. Wu, Ming-Jui Hung, Claire Hung, Nicholas G. Kounis, Ian Y. Chen, Patrick Hu, Chi-Tai Yeh, Ming-Yow Hung

**Affiliations:** 1 The Ph.D. Program for Translational Medicine, College of Medical Science & Technology, Taipei Medical University, Taipei City 11031, Taiwan; chaw1211@tmu.edu.tw; 2 Taipei Heart Institute (THI), Taipei Medical University, Taipei City 11031, Taiwan; 3 Division of Cardiology, Department of Medicine, Chang Gung Memorial Hospital, Keelung, Chang Gung University College of Medicine, Keelung City 20401, Taiwan; miran888@ms61.hinet.net; 4 Kang Chiao International School, Xiugang Campus, New Taipei City 231049, Taiwan; s13435@kcis.com.tw; 5 Department of Cardiology, University of Patras Medical School, 26221 Patras, Greece; ngkounis@otenet.gr; 6 Division of Cardiovascular Medicine, Department of Medicine, Department of Radiology, Stanford Cardiovascular Institute, Stanford University School of Medicine, Stanford, CA 94305-5105, USA; iychen@stanford.edu; 7 Riverside School of Medicine, University of California, Riverside, CA 92521, USA; dr.hu.md@gmail.com; 8 Department of Cardiology, Riverside Medical Clinic, Riverside, CA 92506, USA; 9 Department of Medical Research and Education, Shuang Ho Hospital, Taipei Medical University, New Taipei City 23561, Taiwan; ctyeh@s.tmu.edu.tw; 10 Department of Medical Laboratory Science and Biotechnology, Yuanpei University of Medical Technology, Hsinchu City 30015, Taiwan; 11 Division of Cardiology, Department of Internal Medicine, Shuang Ho Hospital, Taipei Medical University, New Taipei City 23561, Taiwan; 12 Division of Cardiology, Department of Internal Medicine, School of Medicine, College of Medicine, Taipei Medical University, Taipei City 11031, Taiwan

**Keywords:** coronary artery spasm, prevalence, incidence, sex difference, geographic variation

## Abstract

**Background:** Coronary artery spasm (CAS) is a reversible cause of myocardial ischemia, infarction, and sudden cardiac death. Often under-recognized and influenced by ethnicity, geography, and environment, CAS requires population-level data. This study examined nationwide CAS epidemiology in Taiwan by age, sex, region, and season. **Methods:** This cross-sectional population-based study used the Taiwan National Health Insurance Research Database (2004–2018). Diagnosis-coded CAS was operationalized using ICD-9-CM code 413.1 or ICD-10-CM code I20.1 recorded on at least three outpatient encounters or one inpatient encounter. Age- and sex-standardized prevalence and incidence were calculated, and temporal, geographic, and seasonal variations assessed. **Results:** Among 27,974 patients with diagnosis-coded CAS, the mean age at diagnosis was 57.9 years, with nearly equal sex distribution (49.7% female). In 2018, the age-standardized prevalence was 35.1 per 100,000 population, with comparable rates in men (35.4) and women (34.8 per 100,000). The incidence was 11.5 per 100,000 in both sexes. Both prevalence and incidence increased linearly from 2004 to 2018 (*p* < 0.001), peaked at ages 75–79 years, and then declined. Geographic analysis revealed the highest disease burden in central Taiwan, particularly in Taichung City, Changhua County, and Yunlin County. Seasonal analysis showed a clear pattern, with incidence peaking in winter (December–February) and spring (March–May), and notably lower counts in summer and autumn. **Conclusions:** From 2004 to 2018, CAS prevalence and incidence increased in Taiwan, affected both sexes similarly, peaked at ages 75–79 years, and were highest in central western regions during winter and spring. These findings may improve CAS recognition and resource allocation.

## 1. Introduction

Epicardial coronary artery spasm (CAS), a reversible cause of myocardial ischemia characterized by short, severe artery constriction that reduces blood flow, contributes to myocardial infarction, recurrent angina, emergency department utilization, and cardiovascular morbidity in East Asian populations [1]. The prevalence and incidence of CAS are significantly higher in East Asians—notably Japanese, Korean, and Taiwanese—than in Caucasians, with its global prevalence varying by geography, ethnicity, and sex, all of which influence clinical diagnosis and treatment [2]. In patients without obstructive coronary artery disease (CAD), CAS occurs in around 50% of angina (16–73%) [3,4] and 57% of acute coronary syndrome cases [3,5], highlighting the need for routine diagnostic evaluation to prevent misdiagnosis. In CAS patients undergoing coronary arteriography, the prevalences of multiple CASs (≥2 spastic coronary arteries) by provocative testing in Korean (intravenous, 57.1%) [6], Japanese (intracoronary, 24.3%) [7] and Taiwanese populations (intracoronary, 19.3%) [8] are markedly higher than those in Caucasians (intravenous, 7.5%) [9]. Because Asian populations demonstrate 3–4 times higher prevalence rates than the Western World population, more aggressive diagnostic testing in these populations is warranted [1,2]. In 2010, angina from ischemic heart disease affected about 112 million people worldwide (1.6% of the population), being slightly more common in males (1.7%) than females (1.5%) [10]; hence, a 50% prevalence of CAS represents millions of affected individuals globally, highlighting the need for investment in diagnostic facilities and training. The incidence of CAS-induced angina also demonstrates marked geographic and ethnic variations, ranging from 1.7% in the Duke study (USA) [11] to 13% in Shimokawa’s study (Japan) [12], with intermediate rates of 4–5% in the Pisa study (Italy) [12] and 4% in the Montreal study (Canada) [12], reflecting higher prevalence in East Asian compared to Western countries. The incidence of vasospastic angina pectoris was higher in Japan than in Western countries. Additionally, recurrent angina occurs in 10–53% of patients during follow-up, emphasizing the chronic nature of CAS and the necessity for effective long-term management strategies to improve patient quality of life [4]. While there is evidence for racial differences in coronary constrictor response, the prevalence and incidence of CAS in different populations remains to be defined. Therefore, systematic analysis of health system administrative data helps improve our understanding of disease burden by integrating incidence and prevalence data to characterize the epidemiology of coronary artery spasm in Taiwan and to generate evidence relevant to healthcare planning and environmental cardiovascular health policy.

While CAS is common in myocardial ischemia globally, it shows unique sex- and age-related epidemiological patterns that differs notably from atherosclerotic CAD. CAS occurs more frequently in men than women in both East Asian and Western populations [3]. This male predominance suggests a fundamental biological or behavioral difference in the susceptibility to CAS. Most individuals diagnosed with CAS are aged between 40 and 70, and its prevalence usually decreases after 70 years old [3]. The reduction in prevalence after age 70 contrasts sharply with CAD, which becomes more common as people get older. The mechanisms underlying this age-related decline in CAS prevalence remain incompletely understood. Despite the recognition of sex- and age-related patterns in CAS epidemiology, comprehensive population-based studies examining sex- and age-stratified prevalence and incidence across entire populations remain limited. Most existing data derives from selected patient cohorts undergoing coronary arteriography, which may not reflect the true burden of CAS in the general population. We, therefore, conducted a population-based epidemiological study utilizing Taiwanese large health system administrative databases to quantify the population burden of diagnosis-coded CAS and identify demographic, geographic, and environmental patterns relevant to cardiovascular prevention and health-service planning.

## 2. Materials and Methods

### 2.1. Data Source

This was a cross-sectional epidemiology study, which utilized the data from the Taiwan National Health Insurance Research Database (NHIRD). The Taiwan NHIRD contains data for outpatient and inpatient services, including diagnoses, medications, interventions, operations, hospitalizations and emergency visits from the inception of Taiwan’s National Health Institute (NHI) program since March 1995. The NHIRD is currently curated and managed by the Health and Welfare Data Science Center of the Taiwan Ministry of Health and Welfare. The NHI program in Taiwan is obligational, mandatory and universal, and provided approximately 99.8% coverage for the 23.9 million citizens in Taiwan. Diagnoses were recorded based on the International Classification of Diseases, Ninth Revision, Clinical Modification (ICD-9-CM) before 31 December 2015, and then both ICD-9-CM and Tenth Revision (ICD-10-CM) were used thereafter. Comprehensive descriptions of the NHIRD have been documented in the previous literature [13]. Data obtained from the NHIRD are deidentified from all personal information and therefore this study was exempted from obtaining informed consent from patients and was approved by the Taipei Medical University–Joint Institutional Review Board (N202410068; date of approval: 25 November 2025). This study adhered to the principles of the Declaration of Helsinki.

### 2.2. Patient Population

The data analyzed in this study spanned the period from 2000 to 2018. We identified diagnosis-coded CAS patients within the NHIRD—covering the entire Taiwanese population—between 1 January 2004 and 31 December 2018. To ensure the accuracy of the CAS diagnosis, we required patients to have at least three outpatient diagnoses or one inpatient diagnosis (ICD-9-CM: 413.1 and ICD-10-CM: I20.1) during the study period [14] (Supplementary Table S1). To distinguish newly diagnosed CAS, we used 2000–2003 as a washout period and did not include these years for cohort inclusion. Additionally, individuals younger than 20 years or those with missing demographic data were excluded from the analysis.

### 2.3. Estimation of Prevalence and Incidence of Diagnosis-Coded CAS

Prevalent CAS cases were defined as individuals diagnosed with CAS within the 15-year period (2004–2018) prior to December 31 of each calendar year. That is, prevalent cases represented patients who generated at least three outpatient claims or one inpatient claim for CAS during that given year. Incident CAS cases were defined as individuals diagnosed with CAS during 2004–2018 without prior diagnosis, utilizing a history traceable back to January 2000. That is, incident cases represented patients who first met the diagnostic criteria (at least three outpatient claims or one inpatient admission) during 2004–2018, with the initial diagnosis date establishing the index year. The denominator for prevalence/incidence estimation in each calendar year consisted of all Taiwanese individuals aged ≥20 years as of 1 July. Crude annual prevalence and incidence rates were calculated by dividing the number of prevalent and incident cases in a given year, respectively, by the total adult Taiwanese population for that respective year. Furthermore, the annual prevalence and annual incidence rates were standardized according to the 2004 Taiwan population structure, utilizing single-year age groups for the standardization process.

In addition, period prevalence and cumulative incidence were evaluated across various age groups and counties/cities in Taiwan. The numerator for period prevalence was the total accumulated prevalent cases from 2004 to 2018, whereas that for cumulative incidence was the total accumulated incident cases over the same period. For both metrics, the denominator was defined as the average mid-year population between 2004 and 2018. Age groups were categorized in 5-year intervals starting from 20 years of age (i.e., 20–24, 25–29, …), with the oldest category defined as ≥90 years. There were 19 regions on the main island of Taiwan: Taipei city, New Taipei county, Keelung city, Taoyuan city, Hsinchu city, Hsinchu county, Yilan county, Miaoli county, Taichung city, Changhua county, Yunlin county, Nantou county, Chiayi city, Chiayi county, Tainan city, Kaohsiung city, Pingtung county, Hualien county, and Taitung county. To visualize the geographic variations in the period prevalence and cumulative incidence of CAS across these regions, choropleth maps were utilized.

### 2.4. Baseline Characteristics

We additionally extracted baseline characteristics, including age at CAS diagnosis, sex, residential urbanization level (categorized into four tiers), monthly income (tertiles), and comorbidities. Comorbidities comprised diabetes mellitus, hypertension, dyslipidemia, chronic kidney disease, chronic obstructive pulmonary disease, gouty arthritis, hepatitis C virus infection, depression, psychotic disorders, anxiety, stroke, obstructive CAD, acute coronary syndrome (ACS), and coronary revascularization. Stroke, ACS, and coronary revascularization were defined using any inpatient diagnosis or procedure record occurring at any time before the CAS diagnosis date, whereas the remaining comorbidities were identified based on at least two outpatient diagnoses or one inpatient diagnosis within the year preceding the CAS diagnosis. The Charlson’s Comorbidity Index score was also extracted as a measure of the patients’ overall disease burden.

### 2.5. Statistics Analysis

The baseline characteristics between male and female patients were compared using independent sample *t*-test for continuous variable and chi-square test for categorical variable. We calculated the 95% confidence intervals (CIs) for prevalence and incidence based on the Poisson distribution for the observed number of prevalent and incident CAS cases. Temporal trends in CAS prevalence and incidence over the study period were evaluated using Poisson regression models, with the annual number of CAS cases as the response variable, calendar year (2004–2018) entered as a continuous predictor, and the natural logarithm of the corresponding annual population size included as an offset. Formal county-level ecological regression analyses were not performed because county-specific exposure and outcome datasets were not integrated into a unified analytical panel; hence, the environmental interpretations presented in this study should be considered hypothesis-generating and warrant future investigation. The significance level was set at 0.05 with two-sided test. Other statistical analyses were performed using SAS statistical software, version 9.4 (SAS Institute, Cary, NC, USA).

## 3. Results

### 3.1. Patient Characteristics

A total of 27,974 individuals meeting the diagnosis-coded CAS definition were identified within the Taiwan NHIRD between 2004 and 2018. The mean age at diagnosis of CAS was 57.9 years, with a nearly equal sex distribution (13,917 females; 49.7%). The mean age at diagnosis of CAS was 57.9 years, with a nearly equal sex distribution (13,917 females; 49.7%). The majority of patients were diagnosed between the ages of 40 and 70, with the occurrence demonstrating a decline in patients older than 70 years. The most prevalent comorbidity was hypertension (60%), followed by dyslipidemia (32%), anxiety (18.7%), and diabetes (18.4%). Of note, 36% of the patients had a diagnosis of obstructive CAD prior to their CAS diagnosis; additionally, 8.8% had previously experienced ACS, and 5.1% had undergone coronary revascularization. Male patients with CAS exhibited a higher prevalence of obstructive CAD, ACS, and a history of coronary revascularization compared to their female counterparts (Table 1).

### 3.2. Annual Prevalence of Diagnosis-Coded CAS in Taiwan

In 2018, Taiwan had 19,171,861 adult residents, among whom 8570 (0.045%) were identified as prevalent CAS cases, corresponding to an age-standardized annual prevalence of 35.1 per 100,000 population (95% CI, 34.2–36.0) (Table 2).

**Figure 1:**
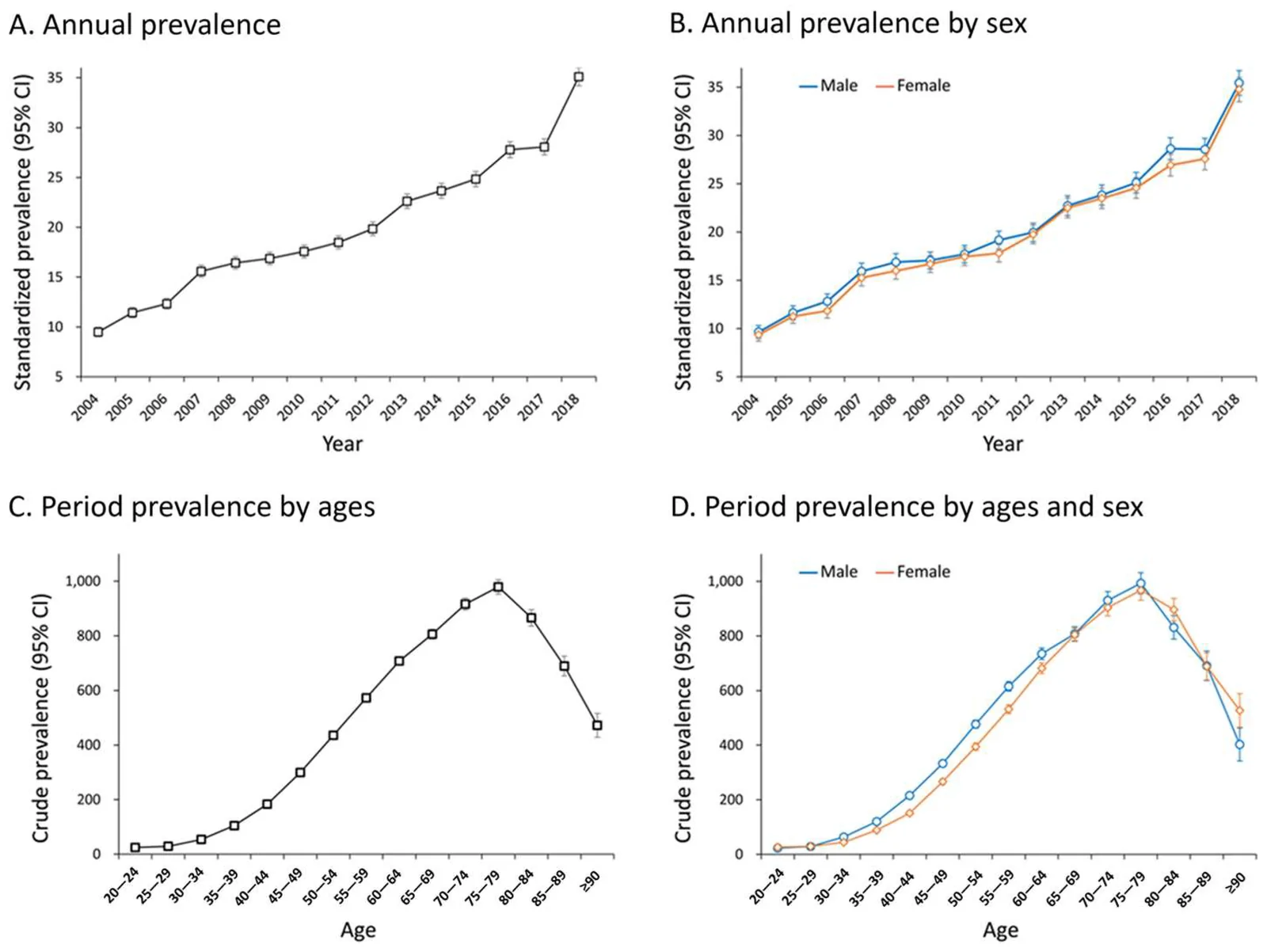
Prevalence of diagnosis-coded coronary artery spasm in Taiwan from 2004 to 2018.

Sex-stratified estimates showed standardized annual prevalences of 35.4 (95% CI, 34.2–36.7) per 100,000 in men and 34.8 (95% CI, 33.5–36.0) per 100,000 in women (Table 3). Figure 1A shows a significant linear increase in CAS annual prevalence in the overall cohort over time (*p* trend < 0.001). When stratified by sex, similar upward trends were observed in both men and women, with no evidence of sex-specific differences (Figure 1B).

The age-specific period prevalence was further calculated (Figure 1C). The results showed an apparent quadratic trend. The period prevalence increased progressively from the 20–24 age group (24.6 cases per 100,000 population, 95% CI 22.2–27.0) to 75–79 years (979.3 events per 100,000 population, 95% CI 952.9–1005.8), at which point it reached a plateau and subsequently declined sharply after the age of 80 (Supplementary Table S2). Figure 1D depicts sex-specific age-stratified period prevalence. In both men and women, period prevalence followed a quadratic pattern across age groups, peaking at 75–79 years.

Annual age-standardized prevalence for the overall population (A) and stratified by sex (B), and age-specific period prevalence for the overall population (C) and stratified by sex (D). The error bar represents the 95% confidence interval of the estimate.

### 3.3. Annual Incidence of CAS in Taiwan

In 2018, out of 19,171,861 adult citizens in Taiwan, 2661 (0.014%) were identified as incident CAS cases, yielding an age-standardized annual incidence of 11.5 (95% CI 11.0–12.0) events per 100,000 population (Table 2). The standardized annual incidence in 2018 was 11.5 (10.8–12.3) for men and 11.5 (10.8–12.2) for women (Table 3).

**Figure 2:**
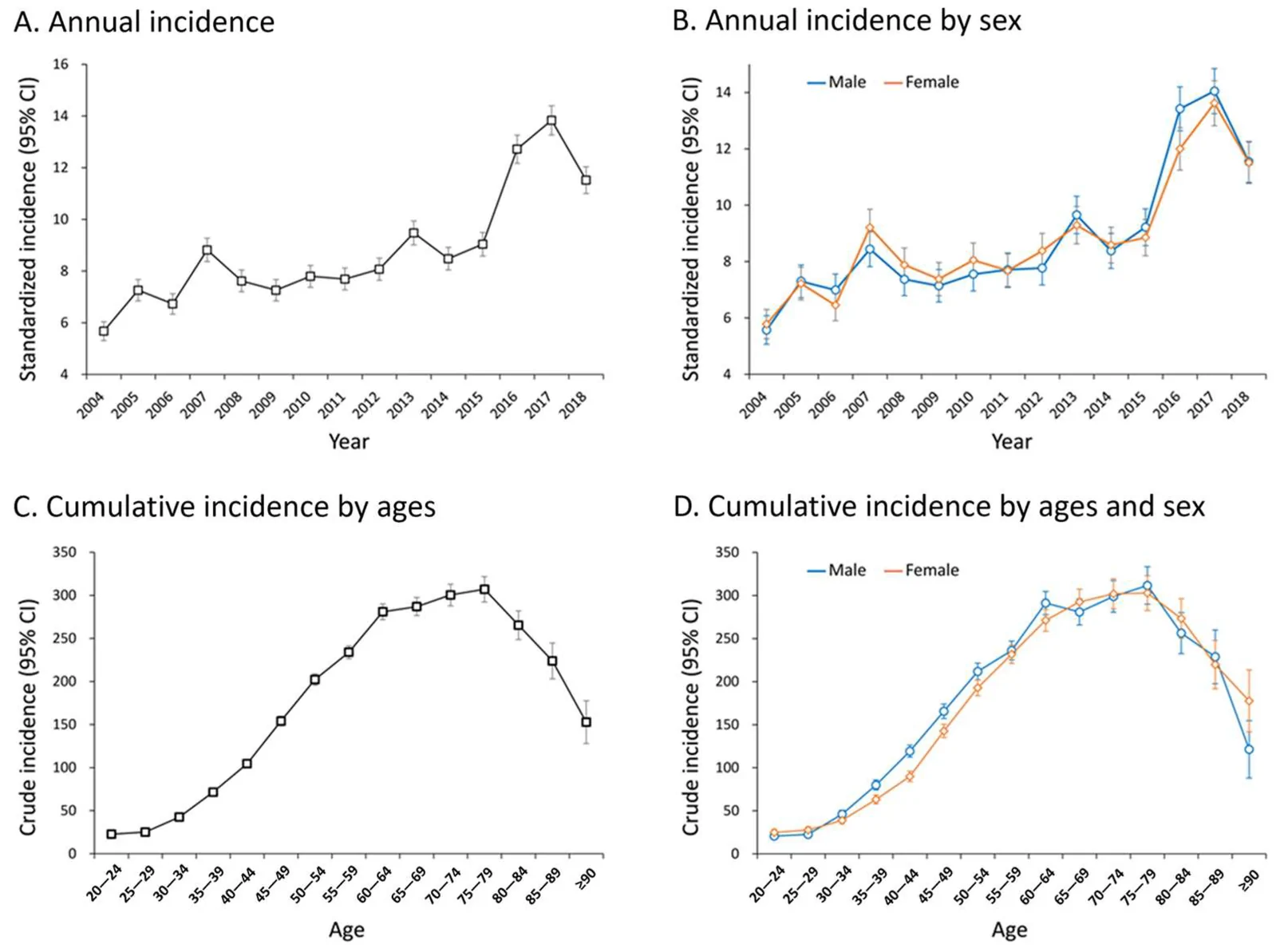
Incidence of diagnosis-coded coronary artery spasm in Taiwan from 2004 to 2018.

Figure 2A demonstrates an increasing linear trend of incident CAS in the whole cohort (*p* trend < 0.001). This linear trend remained consistent across sexes, with no significant sex differences observed (Figure 2B). The crude annual incidence rate rose sharply from 10.50 in 2015 to 15.23 in 2016, coinciding with the nationwide transition from ICD-9 to ICD-10. A Poisson regression model confirmed a significantly elevated incidence rate ratio of 1.78 (95% CI: 1.73–1.83) during the ICD-10 era (2016–2018) relative to the ICD-9 era (2004–2015), underscoring the influence of ICD coding revisions on epidemiological estimates.

Figure 2C further illustrates the age-specific cumulative incidence, which demonstrated a significant quadratic trend. The cumulative incidence rose steadily with age, increasing from 22.6 events per 100,000 population (95% CI, 20.3–24.9) among individuals aged 20–24 years to a plateau at 75–79 years (307.1 per 100,000 population; 95% CI, 292.3–321.9). This peak was followed by a marked decline in those aged ≥80 years (Supplementary Table S3). Figure 2D further illustrates the age-specific cumulative incidence stratified by sex. A significant quadratic trend was observed in both sexes. For both men and women, the cumulative incidence of CAS peaked within the 75–79 years age group.

Annual age-standardized incidence for the overall population (A) and stratified by sex (B), and age-specific cumulative incidence for the overall population (C) and stratified by sex (D). The error bar represents the 95% confidence interval of the estimate.

### 3.4. Geographic Variations in the Period Prevalence and Cumulative Incidence of Diagnosis-Coded CAS in Taiwan

**Figure 3:**
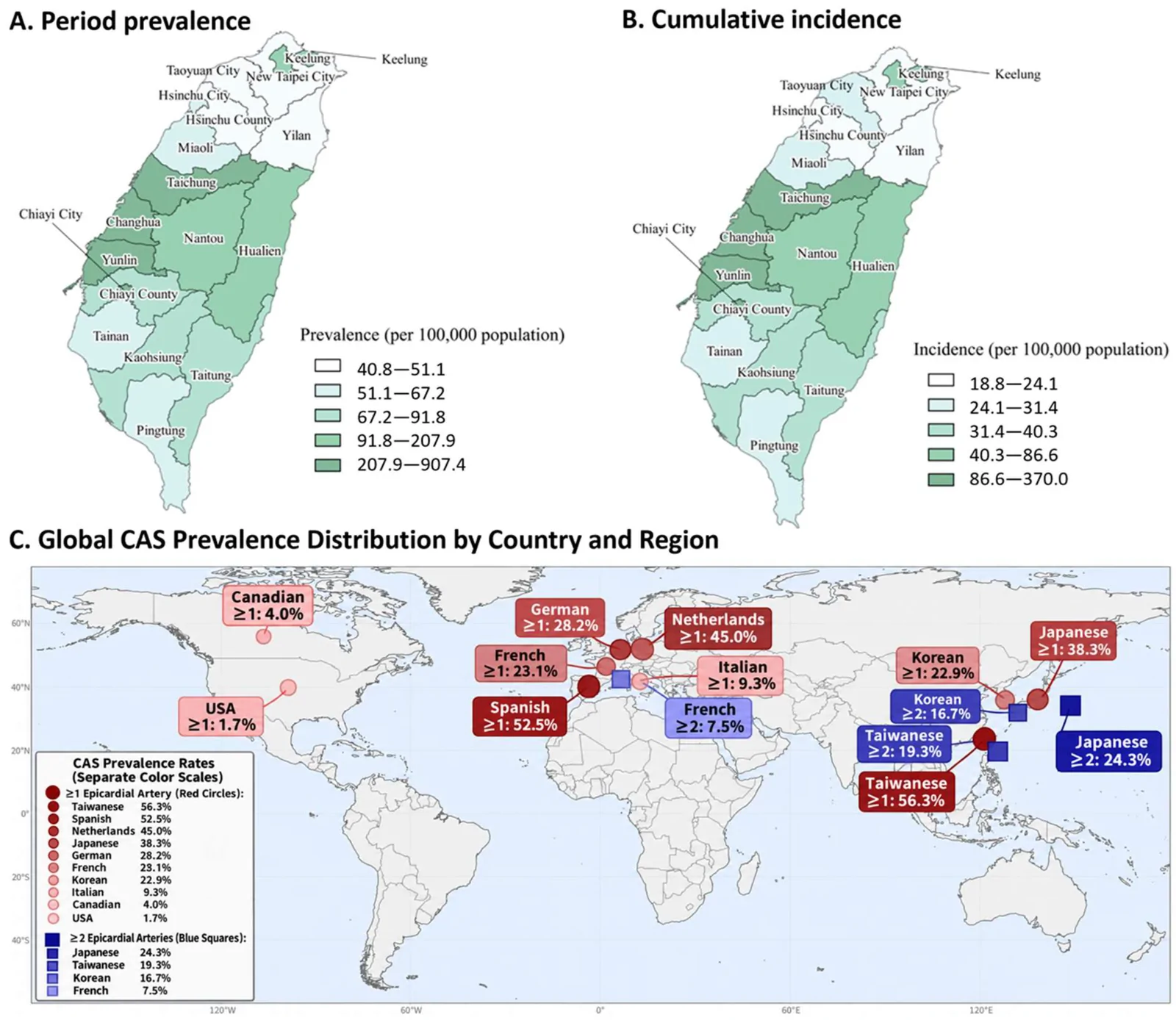
Geographic distribution of diagnosis-coded coronary artery spasm period prevalence and cumulative incidence during 15-year interval. The period prevalence (**A**) and cumulative incidence (**B**) of coronary artery spasm across cities and counties in Taiwan, along with (**C**) a global distribution of coronary artery spasm cohort study reported prevalence by country and region.

We further estimated CAS period prevalence and cumulative incidence across cities and counties in Taiwan. CAS period prevalence was highest in three counties/cities located in central western Taiwan—Taichung City, Changhua County, and Yunlin County (Figure 3A). The geographic pattern of cumulative incidence was broadly similar to that of period prevalence, indicating that CAS was generally more common in central Taiwan (Figure 3B).

### 3.5. Seasonal Variations in the Annual Incidence of CAS in Taiwan

**Figure 4:**
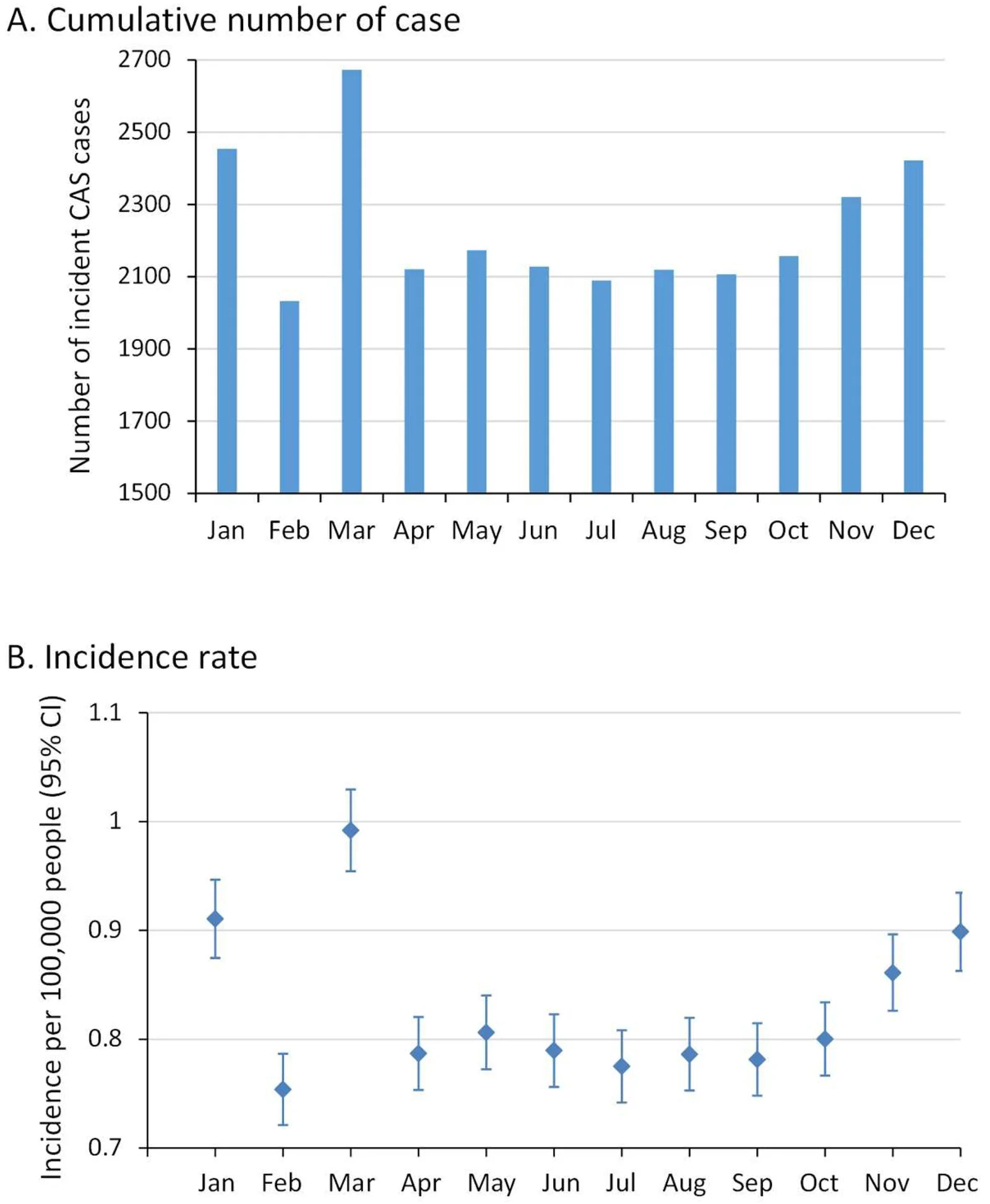
Monthly distribution of incident diagnosis-coded CAS cases (2004–2018). (**A**) Cumulative number of incident cases for each calendar month. (**B**) Monthly incidence rate for each calendar month. The numerator for the monthly incidence rate is the total number of incident cases occurring in that specific month accumulated across the 15-year period, and the denominator is the 15-year summed mid-year population. Error bars represent 95% confidence intervals.

Data for incident CAS from 2004 to 2018 were aggregated and analyzed by the month of initial diagnosis (Figure 4). The results revealed a distinct seasonal pattern: the number of newly diagnosed CAS cases began to rise in November, coinciding with the onset of cooler temperatures, and continued to increase through December and January (the coldest months of the year). A sharp decline was observed in February, followed by a sudden rebound in March, marking the beginning of spring. From April onwards, the annual incidence decreased and remained relatively stable and lower throughout the period leading up to October. We further compared the incidence between cooler months (November through March) and warmer months (April through October). A Poisson regression model confirmed a significantly higher incidence during the cooler months, with an incidence rate ratio of 1.12 (95% CI: 1.09–1.15).

## 4. Discussion

This nationwide population-based study provides the first comprehensive epidemiological characterization of diagnosis-coded CAS in Taiwan, demonstrating that it represents a substantial and growing cardiovascular burden with distinct demographic, temporal, and geographic patterns. In 2018, the age-standardized prevalence and incidence were 35.1 and 11.5 per 100,000 population, respectively, with equivalent rates between men and women. Our findings reveal four critical observations: (1) both prevalence and incidence increased over the study period, (2) disease burden was comparable across sexes, (3) both metrics exhibited a non-linear age distribution with peak incidence at 75–79 years followed by decline, and (4) marked geographic clustering occurred in central western Taiwan, particularly in Taichung City, Changhua County, and Yunlin County, with a distinct seasonal pattern characterized by winter (December to February) and spring (March to May) peaks.

Our age-specific findings both confirm and extend previous work. While CAS is traditionally described as a disorder affecting patients in their 50s [15], and earlier cohort studies suggested a male predominance with a peak between 40 and 70 years [8], our nationwide data demonstrated peak prevalence and incidence at 75–79 years in both sexes [16]. This discrepancy likely reflects methodological differences. Cohort studies often enroll symptomatic patients referred for specialized testing, potentially overrepresenting younger patients with classic presentations. In contrast, nationwide data better capture older adults, atypical presentations, and patients with multiple comorbidities. Consequently, the burden of CAS in older adults may have been underestimated in previous clinical series. Although individuals aged 75–79 years exhibited the highest CAS incidence, the geographic distribution of this elderly population (Supplementary Table S4) did not parallel the distribution of CAS cases across Taiwan. The largest elderly populations were concentrated in metropolitan regions (e.g., New Taipei City, Taipei City, Kaohsiung City), whereas the highest CAS incidence occurred predominantly in central Taiwan (e.g., Taichung City, Changhua County, Yunlin County). This suggests that while advanced age increases susceptibility, population aging alone cannot explain geographic clustering. Instead, aging likely interacts with environmental and regional determinants—such as seasonality, air pollution, urbanization, socioeconomic characteristics, and comorbidity burden—to influence disease occurrence. The decline in incidence after age 80 indicates a non-linear age pattern, differing from atherosclerotic coronary artery disease, which typically increases steadily with age. Possible explanations include survivor effects, competing mortality, changes in autonomic or endothelial function, and under-recognition among the very elderly. This decline should not be interpreted as definitive evidence of a lower biological susceptibility in the oldest age groups. Competing mortality may reduce the opportunity for CAS to be diagnosed because some individuals may die from other causes before receiving a relevant diagnosis. In addition, older adults may have atypical symptoms, reduced healthcare utilization, or lower likelihood of undergoing specialized diagnostic evaluation. Because prevalence and incidence are influenced by both disease occurrence and survival, the lower rate observed at advanced ages may also reflect shorter post-diagnosis survival. The present claims-based data cannot distinguish these mechanisms; therefore, the age-related decline should be regarded as an observed healthcare-recorded pattern that may reflect both biological and ascertainment-related factors. Clinically, CAS should not be regarded solely as a middle-age disorder; it remains a crucial diagnostic consideration in older patients with angina or non-obstructive CAD.

Our results align with the broader literature indicating that CAS is more common in East Asian populations than in Western populations. Higher prevalence has been repeatedly reported in Japanese [7], Korean [6], and Taiwanese [8] cohorts, with meta-analytic data showing epicardial CAS is more frequent in Asian populations (52% vs. 33%) [4]. Prior studies also indicate that CAS accounts for a substantial proportion of angina and acute coronary syndrome cases without obstructive CAD (Table 4) (Figure 3C) [4,6,7,8,9,11,12,17,18,19,20,21,22,23,24,25,26].

Several explanations exist for these ethnic and geographic differences, notably the East Asian aldehyde dehydrogenase 2 (ALDH2) variant, which impairs enzyme function and increases CAS risk [2]. However, genetic predisposition alone is unlikely to explain the full pattern. Environmental exposures, smoking, dietary habits, endothelial dysfunction, vascular reactivity, and healthcare practice differences probably all contribute. These findings reinforce the need for region-sensitive approaches to recognition, diagnosis, and prevention, supporting the view that CAS is a common vasomotor disorder with meaningful implications for clinical care and health-service planning in East Asia.

A significant contribution of this study is its estimation of incidence rates at the population level. Most prior epidemiological work has focused on prevalence, largely because incidence is difficult to determine without clear identification of new cases and valid denominator data. Utilizing a nationwide database with a 4-year washout period helped differentiate new cases from pre-existing conditions. This distinction is vital: prevalence quantifies the total healthcare burden, whereas incidence is more informative for monitoring temporal trends, identifying high-risk populations, and evaluating prevention strategies. Population-based data are particularly valuable here, avoiding selection biases inherent in referral-based cohorts and providing appropriate denominators for public health interpretation [27].

Interpretation of CAS epidemiology remains complicated by the lack of universally applied diagnostic standards. Our case definition relied on International Classification of Diseases codes within a national database—a practical approach for large-scale research but different from studies relying on provocative testing. The angiographic threshold for a positive provocation test varies substantially across the literature (from >50% to >90% luminal narrowing), strongly affecting reported prevalence. While the Coronary Vasomotion Disorders International Study Group (COVADIS) proposed standardized criteria [4], they are not consistently implemented. The COVADIS framework improves specificity but its requirement for ≥90% diameter reduction may miss clinically meaningful cases, including sub-threshold CAS associated with ischemia and symptoms [28]. A strict definition may miss 29.1% of clinically significant CAS, leading to undertreatment. Greater standardization would improve cross-study comparability and our understanding of the global CAS burden.

**Figure 5:**
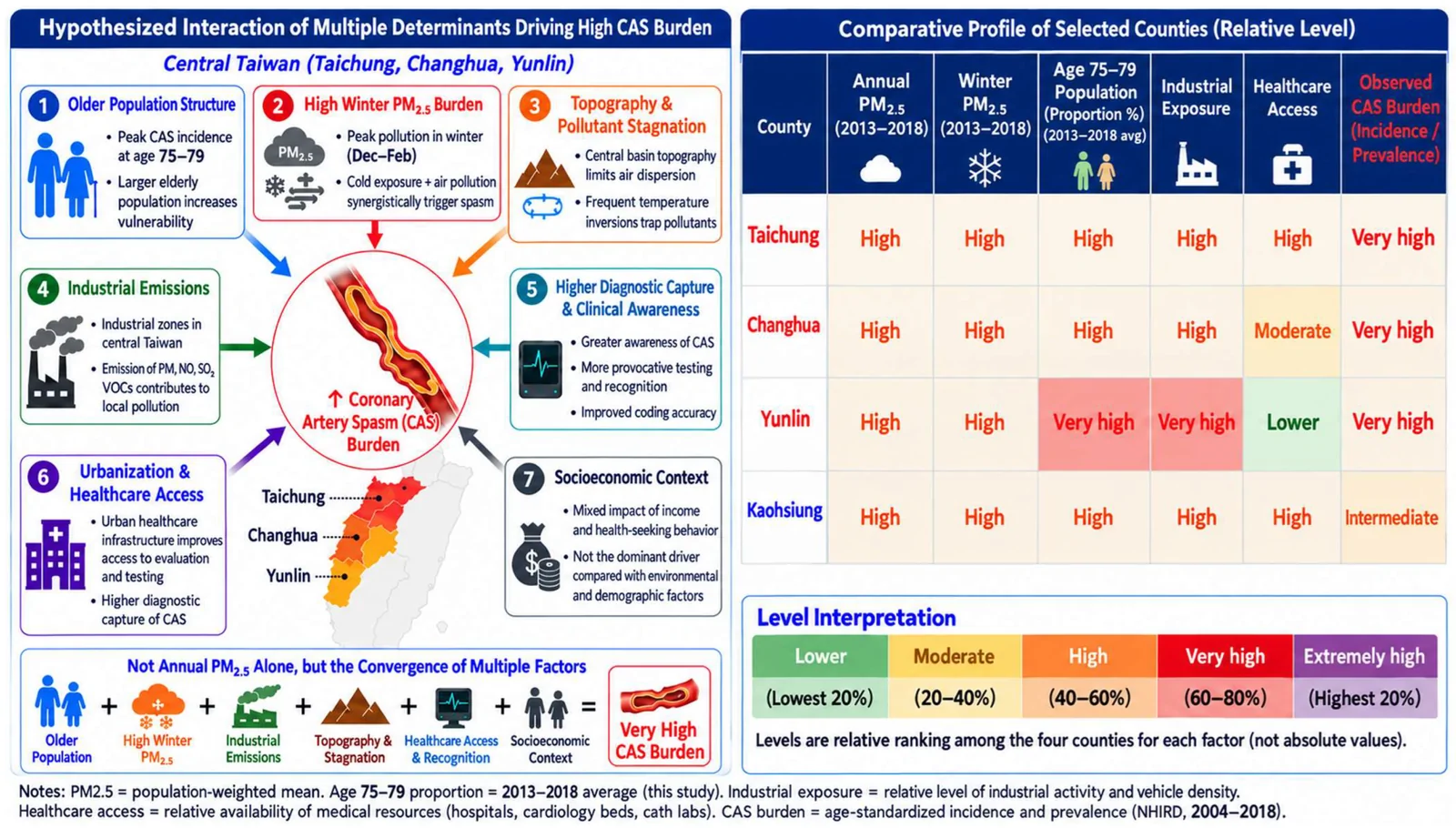
Hypothesis-generating contributors to the higher CAS burden observed in central-western Taiwan. This conceptual schematic summarizes factors that may plausibly contribute to the regional clustering—population aging, ambient and seasonal air pollution, industrial emissions, topographic pollutant stagnation, comorbidity burden, socioeconomic conditions, and healthcare access. These factors were not formally tested in the present study; the figure is intended as a hypothesis-generating framework to guide future individual-level and spatial analyses. PM_2.5_, particulate matter with aerodynamic diameter ≤ 2.5 μm; VOCs, volatile organic compounds; NO_X_, nitrogen oxides; SO_2_, sulfur dioxide.

The geographic clustering of CAS in central-western Taiwan is intriguing and may reflect multiple factors. These regions have historically experienced relatively high levels of ambient air pollution because of industrial activity and unfavorable topography [29,30]. PM2.5 exposure has been associated with both obstructive and non-obstructive myocardial infarction; its stronger association with non-obstructive than with obstructive myocardial infarction suggests a biologically plausible but unconfirmed mechanism, although this evidence remains indirect [31]. Because PM2.5 monitoring was incomplete before 2012 and systematic manual monitoring with official Ministry of Environment reporting began in 2013 (Supplementary Table S5), our study did not link county-level environmental exposures to CAS rates within a unified analytical panel. However, PM2.5 exposure alone based on available county-level ecological data did not explain the observed distribution of CAS. The highest PM2.5 concentrations in Taiwan occur predominantly in southern and southwestern regions, whereas the highest CAS burden was concentrated in central Taiwan. Furthermore, PM2.5 levels in Taiwan peak in winter, driven by transboundary inflow via the northeast monsoon combined with stable atmospheric conditions and Central Taiwan’s topographic pollutant stagnation [32,33]. Emerging evidence supports a biologically plausible link between particulate air pollution and coronary vasomotor dysfunction: exposure to PM2.5 and PM10 has been associated with abnormal coronary vasoconstriction [34], and air pollution reduction has been linked to decreased cardiac morbidity [35]. Regional differences in healthcare access, socioeconomic conditions, physician distribution, and diagnostic awareness may also influence the observed rates [36]. The geographic clustering should therefore be regarded as a hypothesis-generating, multifactorial signal. The CAS burden likely reflects the convergence of multiple regional determinants, including seasonal pollution accumulation, population aging, industrial exposure, healthcare accessibility, and diagnostic capture effects (Figure 5).

The seasonal pattern—with incidence highest in winter and spring—aligns with prior reports of seasonal variation in cardiovascular events [37,38]. A French study reported clear seasonal variation in PM_2.5_ concentrations, with higher levels in winter and lower levels in summer, alongside a winter peak in coronary events [39]. Several other mechanisms may explain the excess burden during colder months: cold exposure activates the sympathetic nervous system [40], lower temperatures impair endothelial function [41], systemic inflammation intensifies [37], and cold conditions increase vascular smooth muscle contractility [40]. The lower incidence observed in February should be interpreted cautiously. In addition to possible meteorological variation, reduced healthcare utilization during the Chinese Lunar New Year holiday and the shorter duration of February may have reduced the number of healthcare-recorded diagnoses. No formal adjustment for the Lunar New Year period was performed because a direct measure of holiday-related healthcare utilization was unavailable in the NHIRD. Therefore, the monthly pattern should not be interpreted as a purely biological or weather-driven effect. Seasonal analyses must consider both biological mechanisms and cultural/administrative influences.

This study has several strengths, notably the use of nationwide data allowing comprehensive assessment of patterns in a large real-world population, and the 4-year washout period strengthening incident case identification. Nonetheless, limitations exist. First, the reported long-term period prevalence likely underestimates true lifetime prevalence due to clinically silent cases. Second, administrative data reflects healthcare encounters rather than symptom onset. Third, we agree that smoking, dietary habits, and genetics are potentially important determinants of coronary vasomotor dysfunction and may contribute to individual susceptibility to CAS. However, these variables were not available in the NHIRD and therefore could not be incorporated into our analyses. As a result, we cannot assess their independent associations with diagnosis-coded CAS, evaluate their contribution to the observed temporal or geographic patterns, or exclude residual confounding related to these factors, leaving important aspects of disease susceptibility and mechanism unresolved. The primary aim of this study was to describe the nationwide epidemiology of healthcare-recorded diagnosis-coded CAS, including its temporal, demographic, geographic, and seasonal patterns, rather than to develop a comprehensive etiologic model. Future studies integrating claims data with clinical registries, standardized lifestyle information, biomarker data, and genetic or biobank resources will be necessary to investigate the biological and behavioral determinants of CAS and to explain the regional differences observed in this study. Fourth, standardized county-level PM2.5 data were not available for the full study period. Taiwan’s automated PM2.5 monitoring network began gradual implementation in August 2005, and nationwide routine manual monitoring was not fully established until December 2012. Therefore, county-level PM2.5 analyses were mainly limited to 2013–2018, when standardized and nationally comparable measurements were available. Consequently, a year-by-year exposure–outcome analysis over the full 2004–2018 period would have been infeasible and would have introduced substantial exposure misclassification, incomplete geographic coverage, and comparability concerns across years. Restricting the analysis to 2013–2018 would also have substantially shortened the observation period and changed the scope of the present study. We have therefore not added a formal pollution–CAS association analysis to the current manuscript. A future dedicated follow-up study integrating year-specific pollution measurements with spatially and temporally matched CAS data, while accounting for demographic structure, healthcare access, and other potential confounders, is planned to address this question directly. Finally, regional variation may partly reflect differences in healthcare access or diagnostic practice rather than true biological risk differences.

## 5. Conclusions

Diagnosis-coded CAS in Taiwan is increasing, affects men and women at similar rates, peaks later in life (75–79 years) than previously suggested, and varies substantially by region and season. These findings refine our understanding of CAS in the general population and underscore the need for greater clinical awareness, standardized diagnostic criteria, and region-specific public health strategies. Better recognition of CAS, particularly in older adults and high-burden regions, is essential for improving clinical outcomes.

## Figures and Tables

**Table 1:** Baseline characteristics of patients with diagnosis-coded coronary artery spasm.

Variable	Total (*n* = 27,974)	Male (*n* = 14,057)	Female (*n* = 13,917)	*p*-Value
Age at diagnosis of CAS (years)	57.9 ± 14.1	57.2 ± 14.0	58.7 ± 14.2	<0.001
Age group				<0.001
20–24	369 (1.3)	175 (1.2)	194 (1.4)	
25–29	441 (1.6)	204 (1.5)	237 (1.7)	
30–34	785 (2.8)	429 (3.1)	356 (2.6)	
35–39	1388 (5.0)	769 (5.5)	619 (4.5)	
40–44	2040 (7.3)	1159 (8.3)	881 (6.3)	
45–49	2947 (10.5)	1570 (11.2)	1377 (9.9)	
50–54	3698 (13.2)	1916 (13.6)	1782 (12.8)	
55–59	3804 (13.6)	1885 (13.4)	1919 (13.8)	
60–64	3735 (13.4)	1871 (13.3)	1864 (13.4)	
65–69	3016 (10.8)	1412 (10.0)	1604 (11.5)	
70–74	2322 (8.3)	1089 (7.8)	1233 (8.9)	
75–79	1763 (6.3)	837 (6.0)	926 (6.7)	
80–84	1054 (3.8)	475 (3.4)	579 (4.2)	
85–89	463 (1.7)	214 (1.5)	249 (1.8)	
≥90	149 (0.5)	52 (0.4)	97 (0.7)	
Urbanization level of the residence				0.412
Low	4748 (17.0)	2347 (16.7)	2401 (17.3)	
Moderate	12,067 (43.1)	6060 (43.1)	6007 (43.2)	
High	7190 (25.7)	3665 (26.1)	3525 (25.3)	
Very High	3969 (14.2)	1985 (14.1)	1984 (14.3)	
Monthly income, NTD$				<0.001
Tertile 1	8271 (29.6)	4097 (29.2)	4174 (30.0)	
Tertile 2	10,133 (36.2)	4722 (33.6)	5411 (38.9)	
Tertile 3	9570 (34.2)	5238 (37.3)	4332 (31.1)	
Comorbidity				
Diabetes mellitus	5132 (18.4)	2542 (18.1)	2590 (18.6)	0.255
Hypertension	16,778 (60.0)	8991 (64.0)	7787 (56.0)	<0.001
Dyslipidemia	8937 (32.0)	4646 (33.1)	4291 (30.8)	<0.001
Chronic kidney disease	2350 (8.4)	1286 (9.2)	1064 (7.7)	<0.001
Chronic obstructive pulmonary disease	2038 (7.3)	1391 (9.9)	647 (4.7)	<0.001
Gouty arthritis	1827 (6.5)	1416 (10.1)	411 (3.0)	<0.001
Hepatitis C virus infection	530 (1.9)	250 (1.8)	280 (2.0)	0.152
Depression	1582 (5.7)	620 (4.4)	962 (6.9)	<0.001
Psychotic disorders	18 (0.1)	9 (0.1)	9 (0.1)	0.983
Anxiety	5232 (18.7)	2086 (14.8)	3146 (22.6)	<0.001
Stroke	1498 (5.4)	826 (5.9)	672 (4.8)	<0.001
Obstructive coronary artery disease	10,135 (36.2)	5749 (40.9)	4386 (31.5)	<0.001
Acute coronary syndrome	2450 (8.8)	1580 (11.2)	870 (6.3)	<0.001
Coronary revascularization	1418 (5.1)	1051 (7.5)	367 (2.6)	<0.001
Charlson’s Comorbidity Index score				<0.001
Mean ± standard deviation	1.1 ± 1.4	1.2 ± 1.5	1.0 ± 1.4	
Median [25th, 75th percentiles]	1 [0, 2]	1 [0, 2]	1 [0, 2]	

Data were presented as frequency (percentage) or mean ± standard deviation. CAS: coronary artery spasm; NTD: New Taiwan Dollar.

**Table 2:** Annual crude and age-standardized diagnosis-coded coronary artery spasm prevalence and incidence, 2004–2018.

	Population	Annual Prevalence Rate *			Annual Incidence Rate *		
Year	Size	Number	Crude (95% CI)	Standardized (95% CI) **	Number	Crude (95% CI)	Standardized (95% CI) **
2004	16,593,129	1577	9.50 (9.03–9.97)	9.50 (9.03–9.97)	942	5.68 (5.31–6.04)	5.68 (5.31–6.04)
2005	16,815,228	1955	11.63 (11.11–12.14)	11.44 (10.93–11.96)	1238	7.36 (6.95–7.77)	7.26 (6.85–7.67)
2006	17,021,654	2171	12.75 (12.22–13.29)	12.33 (11.79–12.86)	1179	6.93 (6.53–7.32)	6.73 (6.33–7.12)
2007	17,214,809	2822	16.39 (15.79–17.00)	15.59 (14.99–16.19)	1589	9.23 (8.78–9.68)	8.82 (8.37–9.27)
2008	17,416,633	3058	17.56 (16.94–18.18)	16.43 (15.82–17.05)	1403	8.06 (7.63–8.48)	7.62 (7.20–8.04)
2009	17,624,651	3234	18.35 (17.72–18.98)	16.87 (16.24–17.49)	1379	7.82 (7.41–8.24)	7.26 (6.85–7.67)
2010	17,826,543	3452	19.36 (18.72–20.01)	17.58 (16.94–18.21)	1507	8.45 (8.03–8.88)	7.80 (7.37–8.22)
2011	18,020,272	3739	20.75 (20.08–21.41)	18.49 (17.83–19.14)	1529	8.48 (8.06–8.91)	7.69 (7.27–8.12)
2012	18,199,752	4115	22.61 (21.92–23.30)	19.85 (19.18–20.53)	1635	8.98 (8.55–9.42)	8.07 (7.64–8.50)
2013	18,379,293	4843	26.35 (25.61–27.09)	22.62 (21.89–23.34)	1977	10.76 (10.28–11.23)	9.48 (9.01–9.94)
2014	18,554,307	5183	27.93 (27.17–28.69)	23.66 (22.92–24.40)	1781	9.60 (9.15–10.04)	8.48 (8.04–8.92)
2015	18,721,556	5613	29.98 (29.20–30.77)	24.84 (24.08–25.59)	1966	10.50 (10.04–10.97)	9.04 (8.58–9.50)
2016	18,883,809	6450	34.16 (33.32–34.99)	27.78 (26.98–28.59)	2876	15.23 (14.67–15.79)	12.72 (12.17–13.26)
2017	19,042,221	6631	34.82 (33.98–35.66)	28.07 (27.27–28.88)	3134	16.46 (15.88–17.03)	13.84 (13.27–14.40)
2018	19,171,861	8570	44.70 (43.75–45.65)	35.11 (34.20–36.01)	2661	13.88 (13.35–14.41)	11.52 (11.00–12.04)

CI: confidence interval. * Number of cases per 100,000 population size. ** Standardized according to the population in 2004 of Taiwan.

**Table 3:** Annual crude and age-standardized diagnosis-coded coronary artery spasm prevalence and incidence by sex, 2004–2018.

	Population	Annual Prevalence Rate *			Annual Incidence Rate *		
Cohort/Year	Size	Number	Crude (95% CI)	Standardized (95% CI) **	Number	Crude (95% CI)	Standardized (95% CI) **
Men							
2004	8,378,305	808	9.64 (8.98–10.31)	9.64 (8.98–10.31)	467	5.57 (5.07–6.08)	5.57 (5.07–6.08)
2005	8,472,284	1001	11.81 (11.08–12.55)	11.63 (10.90–12.36)	627	7.40 (6.82–7.98)	7.30 (6.72–7.88)
2006	8,554,330	1140	13.33 (12.55–14.10)	12.82 (12.05–13.59)	621	7.26 (6.69–7.83)	6.99 (6.43–7.56)
2007	8,628,000	1455	16.86 (16.00–17.73)	15.92 (15.07–16.78)	768	8.90 (8.27–9.53)	8.44 (7.82–9.06)
2008	8,707,835	1582	18.17 (17.27–19.06)	16.88 (16.00–17.76)	681	7.82 (7.23–8.41)	7.37 (6.79–7.95)
2009	8,787,710	1649	18.76 (17.86–19.67)	17.05 (16.17–17.93)	686	7.81 (7.22–8.39)	7.14 (6.57–7.72)
2010	8,864,987	1754	19.79 (18.86–20.71)	17.71 (16.80–18.61)	733	8.27 (7.67–8.87)	7.55 (6.96–8.14)
2011	8,943,954	1950	21.80 (20.83–22.77)	19.16 (18.22–20.09)	770	8.61 (8.00–9.22)	7.71 (7.11–8.30)
2012	9,017,734	2089	23.17 (22.17–24.16)	19.96 (19.00–20.92)	792	8.78 (8.17–9.39)	7.77 (7.17–8.36)
2013	9,092,594	2445	26.89 (25.82–27.96)	22.73 (21.71–23.76)	1006	11.06 (10.38–11.75)	9.65 (8.99–10.32)
2014	9,165,660	2626	28.65 (27.55–29.75)	23.83 (22.79–24.88)	883	9.63 (9.00–10.27)	8.38 (7.76–9.00)
2015	9,235,672	2856	30.92 (29.79–32.06)	25.11 (24.03–26.18)	1006	10.89 (10.22–11.57)	9.22 (8.57–9.87)
2016	9,303,171	3322	35.71 (34.49–36.92)	28.63 (27.49–29.78)	1513	16.26 (15.44–17.08)	13.42 (12.64–14.20)
2017	9,369,319	3363	35.89 (34.68–37.11)	28.56 (27.42–29.71)	1587	16.94 (16.10–17.77)	14.05 (13.25–14.85)
2018	9,421,493	4342	46.09 (44.72–47.46)	35.44 (34.17–36.72)	1337	14.19 (13.43–14.95)	11.54 (10.81–12.26)
Women							
2004	8,214,824	769	9.36 (8.70–10.02)	9.36 (8.70–10.02)	475	5.78 (5.26–6.30)	5.78 (5.26–6.30)
2005	8,342,944	954	11.43 (10.71–12.16)	11.25 (10.53–11.98)	611	7.32 (6.74–7.90)	7.22 (6.64–7.80)
2006	8,467,324	1031	12.18 (11.43–12.92)	11.83 (11.08–12.57)	558	6.59 (6.04–7.14)	6.45 (5.90–7.00)
2007	8,586,809	1367	15.92 (15.08–16.76)	15.26 (14.41–16.10)	821	9.56 (8.91–10.22)	9.21 (8.55–9.86)
2008	8,708,798	1476	16.95 (16.08–17.81)	15.98 (15.11–16.84)	722	8.29 (7.69–8.90)	7.88 (7.27–8.48)
2009	8,836,941	1585	17.94 (17.05–18.82)	16.68 (15.80–17.57)	693	7.84 (7.26–8.43)	7.38 (6.79–7.97)
2010	8,961,556	1698	18.95 (18.05–19.85)	17.44 (16.54–18.35)	774	8.64 (8.03–9.25)	8.05 (7.43–8.66)
2011	9,076,318	1789	19.71 (18.80–20.62)	17.80 (16.89–18.71)	759	8.36 (7.77–8.96)	7.68 (7.08–8.28)
2012	9,182,018	2026	22.06 (21.10–23.03)	19.75 (18.78–20.71)	843	9.18 (8.56–9.80)	8.38 (7.75–9.00)
2013	9,286,699	2398	25.82 (24.79–26.86)	22.50 (21.47–23.52)	971	10.46 (9.80–11.11)	9.29 (8.63–9.95)
2014	9,388,647	2557	27.24 (26.18–28.29)	23.48 (22.44–24.53)	898	9.56 (8.94–10.19)	8.59 (7.95–9.22)
2015	9,485,884	2757	29.06 (27.98–30.15)	24.56 (23.49–25.63)	960	10.12 (9.48–10.76)	8.85 (8.21–9.49)
2016	9,580,638	3128	32.65 (31.51–33.79)	26.92 (25.80–28.04)	1363	14.23 (13.47–14.98)	12.00 (11.25–12.75)
2017	9,672,902	3268	33.79 (32.63–34.94)	27.58 (26.44–28.71)	1547	15.99 (15.20–16.79)	13.62 (12.82–14.42)
2018	9,750,368	4228	43.36 (42.06–44.67)	34.76 (33.49–36.04)	1324	13.58 (12.85–14.31)	11.51 (10.77–12.24)

CI: confidence interval. * Number of cases per 100,000 population size. ** Standardized according to the population in 2004 of Taiwan.

**Table 4:** Prevalence and incidence of CAS: key studies and meta-analyses compared across populations.

Study (Year)	Type	Provocative Route	Epicardial CAS Arteries	Population	Prevalence/Incidence	Notes
Sueda et al. (2004) [7]	Prospective cohort	IC	≥1	Japanese	29.0% prevalence	CAS was defined as >99% diameter reduction. Patients had <75% obstructive CAD.
Odaka et al. (2017) [17]	Prospective cohort	IC	≥1	Japanese	73.0% prevalence	CAS was defined as >90% diameter reduction.
Hung et al. (2010) [8]	Prospective cohort	IC	≥1	Taiwanese	56.3% prevalence	CAS was defined as >70% diameter reduction. Patients had ≤50% obstructive CAD.
Park et al. (2013) [6]	Prospective cohort	IV	≥1	Korean	29.2% prevalence	CAS was defined as >70% diameter reduction. Patients had ≤50% obstructive CAD.
Choi et al. (2019) [18]	Prospective cohort	IC	≥1	Korean	57.6% prevalence	CAS was defined as >75% diameter reduction. No right coronary artery provocation test
Bertrand et al. (1982) [9]	Prospective cohort	IV	≥1	France	23.1% prevalence	CAS was defined as >75% diameter reduction. Patients had ≤50% obstructive CAD.
Coma-Canella et al. (2006) [19]	Prospective cohort	IC	≥1	Spain	52.5% prevalence	CAS was defined as >75% diameter reduction.
ACOVA study (2012) [20]	Prospective cohort	IC	≥1	Germany	28.2% prevalence	CAS was defined as >75% diameter reduction. Patients had ≤20% obstructive CAD.
Jansen et al. (2021) [21]	Prospective cohort	IC	≥1	Netherlands	45.0% prevalence	CAS was defined as >90% diameter reduction. No right coronary artery provocation test
Park et al. (2013) [6]	Prospective cohort	IV	≥2	Korean	16.7% prevalence	Highest reported prevalence in East Asia
Sueda et al. (2004) [7]	Prospective cohort	IC	≥2	Japanese	24.3% prevalence	
Hung et al. (2010) [8]	Prospective cohort	IC	≥2	Taiwanese	19.3% prevalence	Higher than Caucasians, lower than Japanese and Korean
Bertrand et al. (1982) [9]	Prospective cohort	IV	≥2	France	7.5% prevalence	Significantly lower than East Asian populations
Beltrame et al. (1999) [22]	Review	IV/IC	≥1	Japanese/ Caucasians	Japanese: 69-80% prevalence/ Caucasians: 6-37% prevalence	Patients had either variant angina or myocardial infarction. Provocative testing times post-infarction varied across studies. Intracoronary was more sensitive than intravenous.
Woudstra et al. (2023) [4]	Meta-analysis	IV/IC	≥1	ANOCA Patients	Asia: 52% prevalence/ Caucasians: 33% prevalence	
Kyushu (1988) [12]	Prospective cohort	IV	≥1	Japanese	13% prevalence	Patients with ECG ST ↑ included only. CAS was defined as >50% diameter reduction. Higher end of reported incidence rates
Duke (1984) [11]	Prospective cohort	IV	≥1	USA	1.7% prevalence	Patients with ECG ST ↑ included only. Lower end of reported incidence rates
Pisa (1978) [23,24]	Prospective cohort	IV	≥1	Italy	4–5% (-1974) 14% (1974-) prevalence	Patients with ECG ST ↑ included only.
Montreal (1983) [25,26]	Prospective cohort	IV	≥1	Canada	4% prevalence	Patients with ECG ST ↑ included only.

CAD: coronary artery disease; CAS: coronary artery spasm; IC: intracoronary provocation; IV: intravenous provocation; ↑: elevation.

## Data Availability

The data presented in this study are available on request from the corresponding author. The data supporting this article are not publicly available due to information governance restrictions. Access is controlled by the Taiwan Ministry of Health and Welfare and requires prior approval through a formal application process. Researchers interested in obtaining access to the dataset should submit an application to the Ministry and may contact the relevant staff member for assistance at stcarolwu@mohw.gov.tw. Taiwan Ministry of Health and Welfare Address: No. 488, Sec. 6, Zhongxiao E. Rd., Nangang Dist., Taipei City 115, Taiwan (R.O.C.). Phone: +886-2-8590-6848.

## Supplementary Materials

The following supporting information can be downloaded at: https://www.mdpi.com/article/10.3390/medsci14050582/s1, Supplementary Table S1. International Classification of Diseases diagnostic codes (ICD-9-CM: 413.1 and ICD-10-CM: I20.1) used in the study. Supplementary Table S2. Age-specific prevalence of coronary artery spasm from 2004 to 2018. Supplementary Table S3. Age-specific incidence of coronary artery spasm from 2004 to 2018. Supplementary Table S4. Top 10 counties and cities by population aged 75–79 years, 2007–2018. Supplementary Table S5. Mean seasonal PM2.5 concentrations (µg/m^3^) across 19 counties in Taiwan, 2013–2018.

## Abbreviations

CAD: coronary artery disease; CAS: coronary artery spasm; CI: confidence interval; ICD-9-CM: International Classification of Diseases, Ninth Revision, Clinical Modification; ICD-10-CM: International Classification of Diseases, Tenth Revision, Clinical Modification; NHI: National Health Institute; NHIRD: National Health Insurance Research Database.
