# Supplementary material for: Translating Population-Based Epidemiology into Clinical Recognition of Coronary Artery Spasm: Prevalence, Incidence, and Geographic-Seasonal Variation in Taiwan
Source: Med Sci (Basel). 2026 Sep 18;14(5):582. doi: 10.3390/medsci14050582 (PMC13609361; doi:10.3390/medsci14050582)
Supplement: Supplementary file 1 [file medsci-14-00582-s001.zip › medsci-4468231-supplementary.pdf]

Supplementary Materials:

Supplementary Table S1. International Classification of Diseases diagnostic codes (ICD-9-CM: 413.1 and ICD-10-CM: I20.1) used in the study.

Disease	ICD-9	ICD-10
Coronary artery spasm	413.1	I20.1
Diabetes mellitus	250.x	E08-E13
Hypertension	401.x-405.x	I10-I15, N262
Dyslipidemia	272.x	E77, E780, E781, E782, E783, E784, E785, E786, E881, E753, E755, E882, E756, E789, E7521, E7522, E7524, E7130, E7879, E7881, E7889, E8889, E7870
Chronic kidney disease	580.x–589.x, 403.x–404.x, 016.0x, 095.4x, 236.9x, 250.4x, 274.1x, 442.1x, 447.3x, 440.1x, 572.4x, 642.1x, 646.2x, 753.1x,	A1811, D593, E102, E112, E132, I12, I13, K767, M103, M310, N00, N01, N02, N03, N04, N05, N06, N07, N08, N14, N150, N158, N159, N16, N171, N172, N18, N19,

Disease	ICD-9	ICD-10
	283.11, 403.01, 404.02, 446.21	N200, N25, N261, N269, N27, Q61
Chronic obstructive pulmonary disease	491.x, 492.x, 496.x	J41-J44
Gouty arthritis	274.x	M10, M1A (Chronic gout)
Hepatitis C virus infection	070.41, 070.44, 070.51, 070.54, 070.70, 070.71, V02.62	B18.2 (chronic), B17.10 (acute), B17.11 (acute), B19.20 (unspecified), B19.21 (unspecified)
Depression	296.2X, 296.3X, 296.5X, 296.82, 300.4X, 309.0, 309.1, 311, 648.4, 648.44, 780.79	F32, F33
Psychotic disorders	317, 318, 318.1, 318.2, 315.8, 319, 315.39, 315.35, 307.9, 315, 315.1, 315.2, 307.3, 315.4, 299, 299.00, 315.9	F70, F71, F72, F73, F78, F79, F80, F81, F98.4, F82, F84, F88, F89
Anxiety	300.x	F40, F41

Disease	ICD-9	ICD-10
Stroke	430.x–437.x	I60-I62, I66, I65.1, I65.0, I65.8, I65.9, I63.6, I63.8, I63.9, G45.0, G45.8, G45.1, G45.2, G46.0, G46.1, G46.2, G45.9, G45.4, G46.3, G46.4, G46.5, G46.6, G46.7, G46.8, I67.0, I67.1, I67.2, I67.4, I67.5, I67.6, I67.7, I67.9, I68.0, I68.2, I68.8
Coronary artery disease	410.x–414.x, excluding 413.1	I20-I25, excluding I20.1 and I25.111
Acute coronary syndrome	410.x, 411.1, 411.8x, 412.x	I21-I22, I20.0, I24.0, I24.8

ICD: International Classification of Diseases.

Supplementary Table S2. Age-specific prevalence of coronary artery spasm from 2004 to 2018.

	Number of patients with coronary artery spasm per 100,000 population (95% CI)					
	Overall		Male		Female	
Age, year	Population size	Prevalence (95% CI)	Population size	Prevalence (95% CI)	Population size	Prevalence (95% CI)
20–24	1,614,202	24.6 (22.2–27.0)	835,241	23.0 (19.7–26.2)	778,961	26.3 (22.7–29.9)
25–29	1,751,622	28.8 (26.3–31.3)	896,398	28.4 (25.0–31.9)	855,224	29.2 (25.6–32.9)
30–34	1,817,156	53.9 (50.6–57.3)	915,634	63.6 (58.4–68.7)	901,523	44.1 (39.8–48.5)
35–39	1,883,508	104.5 (99.9–109.1)	939,070	120.3 (113.3–127.3)	944,438	88.7 (82.7–94.7)
40–44	1,896,365	183.1 (177.0–189.2)	943,590	215.3 (206.0–224.7)	952,776	151.1 (143.3–158.9)
45–49	1,836,825	299.4 (291.5–307.3)	912,682	333.0 (321.1–344.8)	924,143	266.2 (255.7–276.7)
50–54	1,763,857	435.4 (425.7–445.1)	873,266	477.1 (462.6–491.5)	890,591	394.6 (381.5–407.6)
55–59	1,567,824	572.8 (560.9–584.6)	769,842	615.5 (597.9–633.0)	797,982	531.6 (515.6–547.6)

60–64	1,274,565	707.5 (692.9–722.1)	617,899	734.7 (713.4–756.1)	656,667	681.9 (662.0–701.9)
65–69	990,235	806.1 (788.4–823.8)	471,340	807.9 (782.2–833.6)	518,895	804.4 (780.0–828.8)
70–74	724,377	916.2 (894.2–938.3)	339,598	930.2 (897.8–962.7)	384,778	903.9 (873.9–933.9)
75–79	536,993	979.3 (952.9–1005.8)	252,459	993.0 (954.2–1031.9)	284,534	967.2 (931.1–1003.3)
80–84	371,893	866.1 (836.2–896.0)	173,202	831.4 (788.5–874.3)	198,691	896.4 (854.7–938.0)
85–89	198,293	689.4 (652.8–725.9)	90,467	690.9 (636.7–745.0)	107,825	688.2 (638.6–737.7)
≥90	94,934	471.9 (428.2–515.6)	42,008	402.3 (341.7–463.0)	52,927	527.1 (465.3–589.0)

CI: confidence interval.

Supplementary Table S3. Age-specific incidence of coronary artery spasm from 2004 to 2018.

Age, year	Number of patients with coronary artery spasm per 100,000 population (95% CI)					
	Overall		Male		Female	
	Population size	Incidence (95% CI)	Population size	Incidence (95% CI)	Population size	Incidence (95% CI)
20–24	1,614,202	22.6 (20.3–24.9)	835,241	20.6 (17.5–23.7)	778,961	24.8 (21.3–28.3)
25–29	1,751,622	25.1 (22.7–27.4)	896,398	22.5 (19.4–25.6)	855,224	27.7 (24.2–31.2)
30–34	1,817,156	42.5 (39.5–45.5)	915,634	46.1 (41.7–50.5)	901,523	38.9 (34.9–43.0)
35–39	1,883,508	71.5 (67.7–75.3)	939,070	79.9 (74.2–85.6)	944,438	63.2 (58.1–68.3)
40–44	1,896,365	104.5 (99.9–109.1)	943,590	119.2 (112.3–126.2)	952,776	89.9 (83.9–96.0)
45–49	1,836,825	154.1 (148.4–159.7)	912,682	165.6 (157.2–173.9)	924,143	142.7 (135.0–150.4)
50–54	1,763,857	202.2 (195.6–208.9)	873,266	211.7 (202.1–221.4)	890,591	192.9 (183.8–202.0)
55–59	1,567,824	234.0 (226.4–241.5)	769,842	236.2 (225.3–247.0)	797,982	231.8 (221.3–242.4)
60–64	1,274,565	281.0 (271.8–290.2)	617,899	291.3 (277.9–304.8)	656,667	271.2 (258.6–283.8)
65–69	990,235	287.0 (276.5–297.6)	471,340	280.9 (265.8–296.0)	518,895	292.5 (277.8–307.3)
70–74	724,377	300.5 (287.9–313.2)	339,598	298.9 (280.5–317.3)	384,778	302.0 (284.6–319.4)

75–79	536,993	307.1 (292.3–321.9)	252,459	311.7 (290.0–333.5)	284,534	303.0 (282.7–323.2)
80–84	371,893	265.4 (248.8–282.0)	173,202	256.3 (232.5–280.2)	198,691	273.3 (250.3–296.3)
85–89	198,293	223.9 (203.1–244.7)	90,467	228.8 (197.6–260.0)	107,825	219.8 (191.8–247.8)
≥90	94,934	152.7 (127.9–177.6)	42,008	121.4 (88.1–154.7)	52,927	177.6 (141.7–213.5)

CI: confidence interval.

Supplementary Table S4. Top 10 counties and cities by population aged 75–79 years, 2007–2018.

County / city	2007 (persons/%)	2018 (persons/%)	Change (n; %)	CAGR (%/yr)	vs. national CAGR (pp)	National share (%) 2007 → 2018
1. New Taipei City ▲ share +2.87 pp	57,076/2.6	87,074/2.9	+29,998 +52.6%	+3.91	+2.06	11.65 → 14.52
2. Taipei City ▼ share ▼0.46 pp	65,664/1.9	77,592/1.9	+11,928 +18.2%	+1.53	−0.32	13.40 → 12.94
3. Kaohsiung City ▲ share +0.91 pp	54,538/2.4	72,189/3.2	+17,651 +32.4%	+2.58	+0.73	11.13 → 12.04
4. Taichung City ▲ share +0.52 pp	45,137/2.3	58,302/2.7	+13,165 +29.2%	+2.35	+0.50	9.21 → 9.73
5. Tainan City ▼ share ▼0.83 pp	45,315/2.3	50,456/2.6	+5,141 +11.3%	+0.98	−0.87	9.25 → 8.42
6. Taoyuan City ▼ share ▼0.50 pp	36,101/2.3	41,161/2.1	+5,060 +14.0%	+1.20	−0.65	7.37 → 6.87
7. Changhua County ▼ share ▼0.29 pp	31,967/2.8	37,347/3.3	+5,380 +16.8%	+1.42	−0.43	6.52 → 6.23
8. Pingtung County ▼ share ▼0.32 pp	22,692/2.7	25,833/3.0	+3,141 +13.8%	+1.19	−0.66	4.63 → 4.31
9. Yunlin County ▼ share ▼0.30 pp	22,131/3.3	25,327/3.8	+3,196 +14.4%	+1.23	−0.62	4.52 → 4.22
10. Chiayi County ▼ share ▼0.50 pp	18,156/2.8	19,158/3.1	+1,002 +5.5%	+0.49	−1.36	3.70 → 3.20
National total (19 counties)	490,126	599,500	+109,374 +22.3%	+1.85	reference	100.00 → 100.00

The 10 most populous divisions are shown; the national row aggregates all 19 mainland divisions.

Source: Ministry of the Interior, Taiwan. Household Registration Statistics — Population by 5-year age groups.

CAGR: compound annual growth rate over 11 years (2007–2018); National CAGR: +1.85%/yr (national total: 490,126 in 2007; 599,500 in 2018; net change +109,374, +22.3%); National share: each county's population as a percentage of the all-19-county aggregate; pp: percentage points. Pre-2010 New Taipei City data use former Taipei County; Taichung, Tainan, and Kaohsiung reflect combined former city and county values.

Supplementary Table S5. Mean seasonal PM2.5 concentrations (μg/m³) across 19 counties in Taiwan, 2013–2018.

County	Spring	Summer	Autumn	Winter	Annual Mean
1. Keelung City	19.63	12.68	13.69	19.45	16.36
2. Taipei City	18.9	11.8	13.5	18.7	15.7
3. New Taipei City	20.2	12.3	15.1	21.0	17.2
4. Taoyuan City	23.5	13.2	18.1	25.9	20.2
5. Hsinchu City	21.8	12.9	16.2	23.8	18.7
6. Hsinchu County	22.7	13.4	17.5	25.4	19.8
7. Miaoli County	25.8	14.2	20.1	29.4	22.4
8. Taichung City	25.1	14.7	22.8	31.2	23.5
9. Changhua County	26.0	15.0	23.5	31.2	23.9
10. Nantou County	27.89	14.31	27.19	35.5	26.22
11. Yunlin County	28.4	14.8	26.8	38.6	27.2
12. Chiayi City	30.38	12.59	29.62	44.99	29.4
13. Chiayi County	26.98	12.66	23.59	36.5	24.93
14. Tainan City	28.2	14.5	26.4	39.1	27.1
15. Kaohsiung City	28.8	14.9	27.0	40.3	27.8
16. Pingtung County	24.7	13.8	22.4	33.2	23.5
17. Yilan County	16.03	11.04	11.22	15.6	13.47
18. Hualien County	14.5	10.8	11.9	16.1	13.33
19. Taitung County	12.8	10.2	10.9	14.8	12.18
National mean	23.3	13.15	19.9	28.46	21.2

PM2.5: fine particulate matter with aerodynamic diameter ≤2.5 μm.
Source: Ministry of Environment, Taiwan — national manual PM2.5 monitoring data (AQX_P_10). County-level values are the arithmetic mean of all available monitoring stations within each county, averaged across years. Systematic manual PM2.5 monitoring began in 2013; for 11 counties without sufficient

2013–2014 records, values represent the 2015–2018 mean.
Season definition: Spring = March–May; Summer = June–August; Autumn = September–November; Winter = December–February.
